# [Retracted] Sulforaphane regulates apoptosis- and proliferation-related signaling pathways and synergizes with cisplatin to suppress human ovarian cancer

**DOI:** 10.3892/ijmm.2024.5367

**Published:** 2024-03-19

**Authors:** Shi-Feng Kan, Jian Wang, Guan-Xing Sun

Int J Mol Med 42: 2447-2458, 2018; DOI: 10.3892/ijmm.2018.3860

Following the publication of this paper, the authors realized that they had made an error in assembling the data shown in Fig. 6B on p. 2455, and requested the publication of a corrigendum to rectify this error. However, following an independent investigation of the data published in this paper made by the Editorial Office, it was noted that one set of the immunofluorescence assay images shown in Fig. 4A appeared to be strikingly similar to data appearing in different form in a paper published previously in the journal *BMC Medicine* by different authors at different research institutes [Jing Y-Y, Han Z-P, Sun K, Zhang S-S, Hou J, Liu Y, Li R, Gao L, Zhao X, Zhao Q-D *et al*: Tanshinone IIA reduces SW837 colorectal cancer cell viability via the promotion of mitochondrial fission by activating JNK-Mff signaling pathways. BMC Medicine 10: 98, 2012].

Owing to the fact that the abovementioned data in Fig. 4A had already been published prior to its submission to *International Journal of Molecular Medicine,* the Editor has chosen to decline the authors' request to publish a corrigendum, and decided that this paper should be retracted from the Journal instead. After having been in contact with the authors, they accepted the decision to retract the paper. The Editor apologizes to the readership for any inconvenience caused.

